# Hypoglycaemia and severe *plasmodium falciparum *malaria among pregnant sudanese women in an area characterized by unstable malaria transmission

**DOI:** 10.1186/1756-3305-4-88

**Published:** 2011-05-23

**Authors:** Aziem A Ali, Elhassan M Elhassan, Mamoun M Magzoub, Mustafa I Elbashir, Ishag Adam

**Affiliations:** 1 Faculty of Medicine, Kassala University, Sudan; 2 University of Geizera, Wad Medani, Sudan; 3 Faculty of Medicine, University of Khartoum, Sudan

## Abstract

**Background:**

Pregnant women are more susceptible to severe *Plasmodium falciparum *malaria, which can lead to poor maternal and fetal outcomes. Few data exist on the epidemiology of severe *P. falciparum *malaria in pregnant women.

A hospital-based study was carried out to assess the pattern of severe *P. falciparum *malaria among pregnant women at the Kassala and Medani maternity hospitals, which are located in areas of unstable malaria transmission, in eastern and central Sudan, respectively. Pre-tested questionnaires were used to gather socio-demographic, clinical and obstetrical data. Suitable tests were performed for clinical and biochemical investigations.

**Results:**

Among 222 pregnant women diagnosed with malaria at the two hospitals, 40 (18.0%) women at mean (SD) gestational age of 29.3 (6.7) weeks fulfilled one or more of the WHO criteria for severe *P. falciparum *malaria. These were hypoglycaemia (14; 35.5%), severe anaemia (12; 30%), hypotension (10; 25%), jaundice (9; 22.5%), cerebral malaria (6; 15%), repeated convulsions (4; 10%), hyperparasitaemia (4; 10.0%) and more than one manifestation (9; 22.5%). While the mean (SD) presenting temperature was significantly lower for women presenting with hypoglycaemia [38.2(0.6) versus 38.8(0.7) °C, *P *= 0.04], other clinical and biochemical characteristics were not significantly different among women with different manifestations of severe *P. falciparum *malaria.

**Conclusion:**

Preventive measures for pregnant women such as insecticide-treated bednets and chemoprophylaxis may be beneficial in areas of unstable malaria transmission. Early detection and prompt treatment of severe malaria, especially in pregnant women with hypoglycaemia, are needed.

## Background

Malaria in pregnancy is a major public health problem in tropical and subtropical regions of the world. In Africa, millions of women living in malaria-endemic areas become pregnant each year [1,2]. Malaria in pregnancy contributes to significant maternal and perinatal morbidity and mortality. Each year, more than 500,000 women die during pregnancy or childbirth [1]. Severe malaria is a medical emergency associated with high mortality, especially in cases with multiple organ dysfunction [3]. Cerebral malaria and severe malarial anaemia are two major syndromes causing malaria-related mortality [4]. Children and pregnant women are the most vulnerable groups to the severe form of *P. falciparum *malaria [3]. While much literature and many publications are available on severe malaria in children, few published data exist on severe malaria during pregnancy [5]. Pregnant women are more attractive to the main malaria vector and the disease, including its severe form, than their non-pregnant counterparts [6-8]. Understanding the interactions that underlie the disease and its control should be helpful to investigate the epidemiology of severe malaria. Thus, such study is vital and may be of great interest for providing health planners and caregivers with fundamental guidelines for the implementation of preventive measures. In Sudan, the largest country in Africa, high maternal and perinatal mortality have been observed in different regions, and both malaria and anaemia were the major causes of these high levels of mortality [9-11].

Thus, the present study was conducted at the Kassala and Medani maternity hospitals in Sudan, which are located in areas characterized by unstable malaria transmission [12], and where malaria is a substantial burden affecting pregnant women irrespective of their age or parity [13].

## Methods

**Figure 1:**
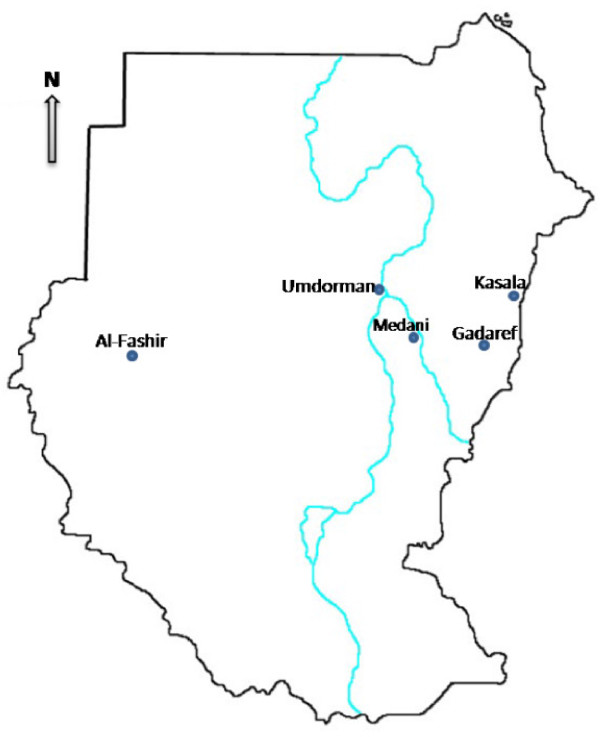
Map showing different regions of Sudan.

This study was conducted at the Kassala and Medani (Figure 1) maternity hospitals in Sudan during the period from July to November 2010 for investigating the epidemiology of severe *P. falciparum *malaria among pregnant women. Pregnant women with symptoms of *P. falciparum *malaria were included in this study after informed consent was obtained from the patient or guardian. Those women with one or more of the manifestations of severe *P. falciparum *malaria according to the World Health Organization (WHO) criteria, which include cerebral malaria (unarousable coma), convulsion (more than two per 24 hours), hypotension (systolic blood pressure < 90 mmHg with cold extremities), severe anaemia (haemoglobin < 7 gm/dl), jaundice (detected clinically or bilirubin > 1 mg/dl), hypoglycaemia (blood glucose < 40 mg/dl) and hyperparasitaemia (parasite count > 100,000 ring forms/μl), were managed according to the WHO guidelines, and the rest were considered as uncomplicated cases [3]. Questionnaires were used to gather socio-demographic, medical and obstetrical data.

Blood films were prepared and stained with Giemsa, and 100 oil immersion fields were examined. The parasite density was evaluated by counting the number of asexual *P. falciparum *parasites for every 200 leukocytes, assuming a leukuocyte count of 8000 leukocytes/μl. All slides were double-checked in a blinded manner and only considered negative if no parasites were detected in 100 oil immersion fields. If gametocytes were seen, then the count was extended to 500 oil immersion fields. Haemoglobin concentrations were estimated using a HemoCue haemoglobinometer (HemoCue AB, Angelhom, Sweden). Blood glucose was measured at baseline before quinine infusion, two hours after quinine infusion and if there was clinical suspicion of hypoglycaemia using the bedside device Accu-Chek™ Multiclix (Roche diagnostics, Mannheim Germany). The Accu-Chek™ machine was calibrated weekly and every time a new box of test strips was opened.

Resuscitation and supportive management were given according to the WHO guidelines [3]; i.e. quinine infusion at 10 mg/kg three times a day over 2-3 hours changed to oral quinine tablet when the patient could tolerate them, correction of hypoglycaemia with 10% glucose, termination of convulsions with intravenous diazepam if they were persisting for more than three minutes. Paracetamol was given every 6 hours until defervescence. Those with severe anaemia (haemoglobin < 7 g/dl) and respiratory distress were transfused with blood screened for hepatitis and HIV. Vital signs were measured every 15 minutes for the first hour, then every 2 hours until 24 hours, and thereafter every 6 hours until the discharge from the hospital. Baseline investigations were performed for every patient on admission and repeated when clinically indicated. These included levels of haemoglobin, serum urea, serum creatinine, and serum bilirubin as well as the white blood cell count.

## Statistics

Data were entered into a computer database and SPSS software (SPSS Inc., Chicago, IL, USA) and double checked before analysis. Means (SD) and proportions for the socio-demographic and biochemical variables were calculated. ANOVA was used to compare the means (SD) between the different groups of severe malaria. Correlations between the different continuous clinical and biochemical variables were calculated. *P *< 0.05 was considered significant.

## Ethics

The study received ethical clearance from the Research Board at the Faculty of Medicine, University of Khartoum.

## Results

**Figure 2:**
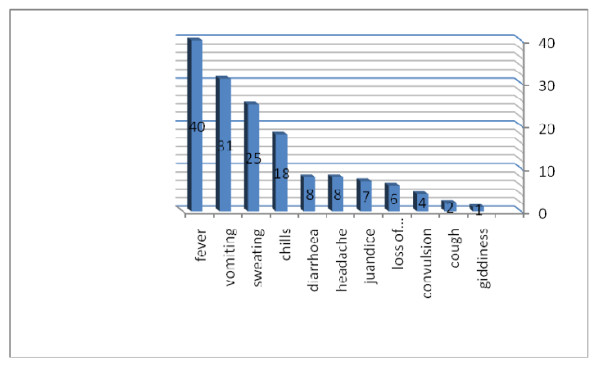
**Presenting symptoms among pregnant women with severe *P. falciparum *malaria**.

Among 222 pregnant women diagnosed with malaria at the two hospitals, 40 (18.0%) fulfilled one or more of the WHO criteria for severe *P. falciparum *malaria. These were hypoglycaemia (14; 35.5%), severe anaemia (12; 30%), hypotension (10; 25%), jaundice (9; 22.5%), cerebral malaria (6; 15%), repeated convulsions (4; 10%), hyperparasitaemia (4; 10.0%) and more than one manifestation (9; 22.5%), Table 1. Ten patients with severe anaemia received blood transfusion. Out the 12 patients with severe anaemia; three patients (25%) had jaundice, one patient had hypotension and hyperparasitaemia (parasite count was 133333 rings/μ) and one patient had hypogylcaemia. The mean (SD) of the age, gravidity and gestational age of these 40 women were 28.4 (6.1) years, 3.5 (2.3) and 29.3 (6.7) weeks, respectively, Table 2. The parity ranged from 1 to 9, (median = 2) and 10 (25%) of these women were primigravidae. None of the patients had used antimalarial chemoprophylaxis. There were no maternal deaths. All women were febrile. Different symptoms such as sweating, aches, vomiting and diarrhoea were observed among these women, Figure 2. None of the patients developed hypoglycaemia during quinine treatment. All patients started oral quinine tablet within two days.

**Table 1:** presentations of severe *P. falciparum *malaria at Kassala and Medani Maternity Hospitals, Sudan

Criteria of severe *P. falciparum *malaria	Number of women	Percentage
Hypoglycaemia	14	35.5
Severe anaemia	12	30.0
Hypotension	10	25.0
Jaundice	9	22.5
Cerebral malaria	6	15.0
Convulsions	4	10.0
Hyperparasitaemia	4	10.0
More than one complication	9	22.5

**Table 2:** presenting clinical and biochemical data of the pregnant women with severe *P. falciparum *malaria at Kassala and Medani Maternity Hospitals, Sudan

Variables	range, mean ± SD
Age, years	28.4 ± 6.1 [18--40]
Gravidity	3.5 ± 2.3 [1--9]
Gestational age, weeks	29.3 ± 6.7 [14--38]
Duration of illness, days	2.5 ± 1.1 [1--6]
Weight, kg	59.3 ± 7.1 [43--75]
Temperature, °c	38.5 ± 0.7 [37.8--40.0]
Haemoglobin, gm/dl	8.3 ± 1.7 [5--11.0]
White blood cells, cell/mm^3^	6300 ± 2927 [2700--14000]
Parasite count, rings/μl	30717 ± 5270 [2080--335242]
Blood glucose, mg/dl	71.4 ± 3.0 [29--120]
serum bilirubin, mg/dl	2.0 ± 0.7 [1--3]
Serum creatinine, mg/dl	1.0 ± 0.2 [0.7--2.0]

While the mean (SD) presenting temperature was significantly lower in women presenting with hypoglycaemia [38.2(0.6) versus 38.8(0.7) °C, *P *= 0.04], other clinical and biochemical characteristics were not significantly different among women with different manifestations of severe *P. falciparum *malaria, Table 3. The haemoglobin level was significantly lower in the anaemic group, Table 3. There was no significant correlation between blood glucose levels and temperature, haemoglobin levels and parasite counts, Table 4.

**Table 3:** comparison of clinical and biochemical data in the subgroups of the pregnant women with severe *P. falciparum *malaria at Kassala and Medani Maternity Hospitals, Sudan

Variable	Hypoglycemic group (*N *= 14)	Anaemic group (*N *= 12)	Other group (*N *= 14)	*P*
Age, years	27.1(8.1)	30.1(5.9)	28.8(4.4)	0.1
Gravidity	3.8(2.9)	3.6(2.7)	3.5(1.9)	0.3
Gestational age, weeks	26.9(6.4)	29.2(8.4)	32.0(4.5)	0.6
Weight, kg	59.0(6.6)	61.3(7.7)	58.3(7.6)	0.2
Temperature, °C	38.2(0.6)	38.5(0.5)	38.8(0.7)	0.04
Haemoglobin, gm/dl	8.4(1.4)	6.2(0.7)	9.2(1.1)	0.001
parasite count, ring/μ	32057(62169.1)	27191(37961)	29244(3633)	0.9

**Table 4:** Correlation between various clinical and biochemical measures including haemoglobin

Variable	Temperature		Blood glucose		Parasite count	
	r	*P*	r	*P*	r	*P*
Haemoglobin	0.242	0.1	0.146	0.3	0.129	0.4
Temperature			0.290	0.07	0.013	0.9
Blood glucose					0.079	0.6

## Discussion

The main findings of the present study were that hypoglycaemia and severe anaemia were the predominant presenting manifestations of severe *P. falciparum *malaria observed during pregnancy in this epidemiological setting. With the exception of lower temperatures, other clinical and biochemical criteria were not different among women with different criteria of severe *P. falciparum *malaria. Previously, severe anaemia and jaundice had been observed as the presenting manifestations of severe *P. falciparum *malaria in pregnant women in central and eastern Sudan [5,14]. In neighbouring Ethiopia, cerebral malaria, convulsions, altered mental state and prostration were the common manifestations of severe malaria observed in pregnant women [15]. Generally, pregnant women are more susceptible to severe malaria and hypoglycaemia than their non-pregnant peers [3,7]. The glucose metabolism during malaria infection is affected by several factors, including drug treatment, fever, parasite metabolism, hormonal changes, cytokines, fasting and gastrointestinal disturbances [16,17]. It has been reported that patients with severe malaria-induced hypoglycaemia have higher mortality rates [18]. Thus, the recognition of patients with falciparum malaria and hypoglycaemia by blood glucose estimation at the time of admission could significantly affect the ultimate outcome. Interestingly, some comatose patients regained consciousness with intravenous fluid infusion of 25% dextrose only without receiving any specific antimalarial treatment [18]. None of these patients developed hypoglycaemia after quinine treatment. In this study blood glucose was investigated at base line, two hours following quinine and if hypoglycaemia was clinically suspected. Ideally, blood glucose should be investigated every four hours if possible especially in comatose patients according to the WHO guidelines [19]. Therefore, this is one of the limitations of this study where quinine -induced hypoglycaemia was not investigated as should be. Previously, only one out of 33 pregnant Sudanese women developed hypoglycaemia following quinine treatment for severe *P. falciparum *malaria [5]. Hyperinsulinaemic hypoglycaemia is the most important adverse effect in the quinine treatment of severe malaria which is particularly common in pregnancy (50% of quinine-treated women with severe malaria in late pregnancy) [20,21]. Intravenous artesunate is superior to quinine in the treatment of severe malaria [22]. Compared to intravenous quinine, intravenous artesunate has been shown to have; a lower risk of hypoglycaemia, significantly reduce the risk of death from severe malaria, and it is not requiring rate controlled infusion or cardiac monitoring [19]. Patients in this series were in their second and third trimester of pregnancy; therefore intravenous artesunate would have been given to these women instead of quinine. However, intravenous artesunate is not yet registered and available in Sudan.

In the present study, 12 (30%) and 6 (15%) patients presented with severe anaemia and cerebral malaria, respectively. Cerebral malaria and severe malarial anaemia are two major syndromes causing malaria-related mortality [4]. The pattern of these two severe forms varies depending on the intensity of transmission; cerebral malaria is more common in older children in areas with lower intensity of transmission, whereas severe malarial anaemia is often seen in children below two years of age in areas with intense transmission [4]. Maternal anaemia and malaria have been reported in areas of unstable malaria transmission in Thailand and in Ethiopia, as well as in areas of stable malaria transmission [23,24]. Regardless of the transmission level and the level of pre-pregnancy immunity against malaria, maternal anaemia remains the most frequent consequence of malaria during pregnancy [25]. Interestingly, we have recently observed a high prevalence of anaemia in pregnant women in these two hospitals and anaemic women were at a higher risk of stillbirth and low birth weight deliveries [26-29]. Interestingly, seven out 12 patients in the current study had severe anaemia without evidence of multiorgan dysfunction or other manifestations of severe of malaria. Although, these patients fulfilled the WHO criteria for severe malaria [3], perhaps some of these women had severe anaemia and concurrent uncomplicated malaria rather than severe *P. falciparum *malaria. Thus, in such situation these patients would have received blood transfusion and artemisinins combination therapy rather than quinine treatment.

There were no maternal deaths in this study, early diagnosis; prompt effective treatment could explain this observation. These women would appear to represent a milder spectrum of disease e.g. severe anaemia and hypotension. It have been shown that, within the broad definition of severe *P. falciparum *malaria there are syndromes associated with mortality rates that are lower (e.g. severe anaemia) and higher (cerebral malaria and metabolic acidosis) [19]. We previously observed that malaria was one of the main causes of high maternal mortality in these two hospitals [9,30]. Maternal mortality is approximately 50% in pregnant women with severe *P. falciparum *malaria, which is higher than in non-pregnant adults [19]. The other limitation of this work is that we could not follow up these women and investigate/report the maternal and perinatal outcomes, and compare them to women with uncomplicated *P. falciparum *malaria and healthy controls.

## Conclusion

Preventive measures for pregnant women such as insecticide-treated bednets and chemoprophylaxis may be beneficial in areas of unstable malaria transmission. Early detection and prompt treatment of severe malaria, especially in pregnant women with hypogylcaemia, are needed.

## Competing interests

The authors declare that they have no competing interests.

## Authors' contributions

AAA and EME carried out the study and participated in the statistical analysis and procedures. MMM carried out the biochemical tests. IA and MIE coordinated and participated in the design of the study, statistical analysis and the drafting of the manuscript. All the authors read and approved the final version.
